# Bibliometric Analysis of Nature-Based Therapy Research

**DOI:** 10.3390/healthcare11091249

**Published:** 2023-04-27

**Authors:** Yeray Rodríguez-Redondo, Angel Denche-Zamorano, Laura Muñoz-Bermejo, Jorge Rojo-Ramos, Jose Carmelo Adsuar, Antonio Castillo-Paredes, Alejandro Vega-Muñoz, Sabina Barrios-Fernandez

**Affiliations:** 1 Social Impact and Innovation in Health (InHEALTH) Research Group, University Centre of Mérida, University of Extremadura, 06800 Mérida, Spain; 2 Promoting a Healthy Society Research Group (PHeSO), Faculty of Sport Sciences, University of Extremadura, 10003 Caceres, Spain; 3 Physical Activity for Education, Performance and Health, Faculty of Sport Sciences, University of Extremadura, 10003 Caceres, Spain; 4 Grupo AFySE, Investigación en Actividad Física y Salud Escolar, Escuela de Pedagogía en Educación Física, Facultad de Educación, Universidad de Las Américas, Santiago 8370040, Chile; 5 Instituto de Investigación y Postgrado, Facultad de Ciencias de la Salud, Universidad Central de Chile, Santiago 8330507, Chile; 6 Public Policy Observatory, Universidad Autónoma de Chile, Santiago 7500912, Chile; 7 Occupation, Participation, Sustainability and Quality of Life (Ability Research Group), Nursing and Occupational Therapy College, University of Extremadura, 10003 Cáceres, Spain

**Keywords:** bibliometrics, health, wellbeing, green spaces, one health

## Abstract

Unrestrained urbanisation and natural space loss are reducing contact with nature in today’s society, producing negative consequences for people’s mental and physical health and wellbeing. Nature-based therapies, such as physical activity in natural settings, forest bathing, therapeutic hiking, or experiential learning, reduce anxiety and depressive symptoms and improve the quality of life in both general and specific populations. A bibliometric analysis of research on nature-based therapies was performed by applying the traditional laws of bibliometrics (exponential growth law, Bradford’s concentration law, Lotka’s law, Zipf’s law, etc.) to documents published in journals indexed in the Core Collection of the Web of Science (WoS). Graphical visualisation was performed using the VOSviewer software. Annual publications between 2006 and 2021 presented an exponential growth trend (R^2^ = 91%). *The International Journal of Environmental Research and Public Health* (MDPI) and *Urban Forestry & Urban Greening* (Elsevier) were the most productive and cited journals. Ikei, Miyazaki, and Song are the most cited prolific authors. The USA and South Korea were the countries with the highest scientific production. In recent years, there has been a growing interest in adventure, nature, and forest therapies among researchers. Nature-based therapies have experienced a growing interest in recent years. Positive effects on mental, physical, and emotional health have been found in different populations and research lines, although more studies with different designs and populations are needed.

## 1. Introduction

At least 32,300 hectares of forest disappear every day, and it is estimated that in the last 40 years, the size of the world’s wildlife has been reduced by 60%; global ecosystems and natural environments have suffered a catastrophic degradation over the last 50 years [1], which is a side effect of fast urbanisation, human expansion, and natural resource exploitation, resulting in societies with reduced possibilities for contact with nature, affecting the population’s global health and quality of life [2,3,4]. Since their origins, humans have adapted to their natural settings, as their survival depends on this [4]. Today’s societies have ever-expanding, highly urbanised and artificial settings, reducing the planet’s natural spaces and leading to lifestyles disconnected from nature, resulting in a greater prevalence of sedentary behaviour, stress, and physiological discomfort [3,4,5,6,7]. Moreover, technology abuse and longer screen time are associated with non-adaptive behaviours, anxiety, and depressive symptoms [5]. Some authors have proposed trying to recover this nature–human connection [5], as the physiological benefits of natural stimuli [4] in health and wellbeing have been widely documented [3,6], although there is still no theoretical development of the processes and mechanisms of change that contribute to the therapeutic results [8,9,10,11].

Nature-based therapies are planned therapeutic techniques performed in natural spaces and based on nature–human active participation and connection [8]. There are various modalities such as forest therapy with or without guidance [3,6,12,13], forest bathing or shinrin-yoku [14,15,16], therapeutic mountain hiking [6], expedition-based therapies, wilderness therapy [17,18,19,20], or walking and talking in natural open spaces [21,22]. Other related therapies, as they can be carried out both in urban or natural settings, are garden therapy [23,24], horticulture [7,25] or animal-based therapy [26,27]. Landscape therapies highlight [28] colour classifications and adopt a holistic approach including, within the “blue therapy” umbrella, coastal zone landscapes [29,30], as well as “green therapy”, such as forests, gardens, and other green spaces [28]. Regardless of the technique, six key elements can be identified in the implementation of nature-based interventions: physical activity, body and mind reconnection, nature metaphors, relationship establishment, natural interaction observations, and experiential learning [31,32,33]. These approaches are considered to be useful in both overall and specific populations, revealing positive physiological, social, emotional, physical, and psychological outcomes [11,34,35,36]. Regarding the positive effects of nature-based therapy on healthy behaviours, physical activity levels have increased in people with mental disorders [33], older adults with depression [32], children and adolescents [36], people with cancer [37] and young adult cancer survivors [38], among others. Moreover, decreased depression, anxiety, anger and confusion symptomatology has been associated with outdoor physical activity [39,40,41]. Furthermore, quality of life benefits have also been reported in people with chronic pain [42], as well as in psychological distress and sleep quality in people with or surviving cancer [37,38].

A bibliometric study is a quantitative technique used to assess scientific production and its impact on a particular field of knowledge. It is based on the analysis of scientific publications and their citations, providing a detailed view of the research state in a specific area based on the analysis of scientific publications and the citations they receive. Thus, such studies can help identify research trends; help researchers make informed decisions; and assess researchers, institutions, and countries’ performance in terms of scientific production and impact [43]. To the best of our knowledge, only one bibliometric study on forest therapies or forest bathing exists but using a different methodology [44]. Furthermore, no bibliometric analysis of nature-based therapies has been conducted in a broad sense, nor have there been any that use traditional bibliometric laws. This study aims to assess publications related to therapies in natural settings identifying the most important journals, the prolific and prominent authors, the most relevant documents, and the most frequently used keywords.

## 2. Materials and Methods

### 2.1. Design and Data Source

A descriptive bibliometric analysis was performed based on publications whose journals were indexed in the Web of Science (WoS) from Clarivate Analytics. The WoS database is a popular resource among researchers for bibliometric analyses, as it includes a significant number of high-quality indexed journals and offers comprehensive information on each publication [43,45,46,47]. The search was conducted on the WoS Core Collection Database, limited to articles and reviews in the Science Citation Index Expanded (SCI-Expanded), Social Sciences Citation Index (SSCI), and Emerging Sources Citation Index (ESCI) editions. For this search, the tag “TI” was used, locating the search terms only in the title of the manuscripts. An advanced search was conducted on 12 September 2022, using the following search vector: ((ti = (“therapy”) or ti = (“psychotherapy”) or ti = (“therapeutic”)) AND (ti = (“wilderness”) OR ti = (“adventure”) OR ti = (“nature”) OR ti = (“outdoor”) OR ti = (“forest”) OR ti = (“garden”))) OR (ti = (“green therapy”) OR ti = (“blue therapy”) OR ti = (“green psychotherapy”) OR ti = (“blue psychotherapy”) OR ti = (“green therapeutic”) OR ti = (“blue therapeutic”)). Therapies that were not necessarily in nature, such as animal-based ones or horticulture, were excluded. Data were exported from WoS in “.xslx” and plain text (“.txt”) formats for further processing in Microsoft^®^ Excel^®^ for Microsoft Office Professional Plus 2019 and VoSViewer.

### 2.2. Data Analysis

A descriptive analysis was performed to assess the trends followed by the annual publications. DeSolla Price’s law of exponential growth of science was applied [48,49], estimating the coefficient of determination (R^2^) adjusted to an exponential growth rate to determine whether annual publications were in an exponential growth phase. The WoS Analyse Reports tool was used to descriptively analyse the WoS categories to which the documents belonged. Bradford’s law of concentration was applied to highlight the journals with the highest number of publications on the topic and those accumulating the highest number of citations [50,51,52,53,54]. Lotka’s law [55] was applied to identify the most productive co-authors in the field, called prolific authors, and on these, the Hirsch index (h-index) was used to reveal the most cited co-authors, with those with the highest number of citations being considered the most productive ones. The prolific co-authors who presented at least one document among the most cited papers were considered prominent co-authors [56,57,58]. The h-index was applied to highlight the most relevant documents, considering n articles with n or more citations [57,58]. The authors’ keywords of most interest were identified by applying Zipf’s law to the set of keywords in the publications from the dataset [59]. VOSviewer was used with a strength of association analysis to create graphs representing the relationships between journals, prominent co-authors, regions/countries, keywords, and the most relevant manuscripts.

## 3. Results

### 3.1. Annual Publication Trends

**Figure 1:**
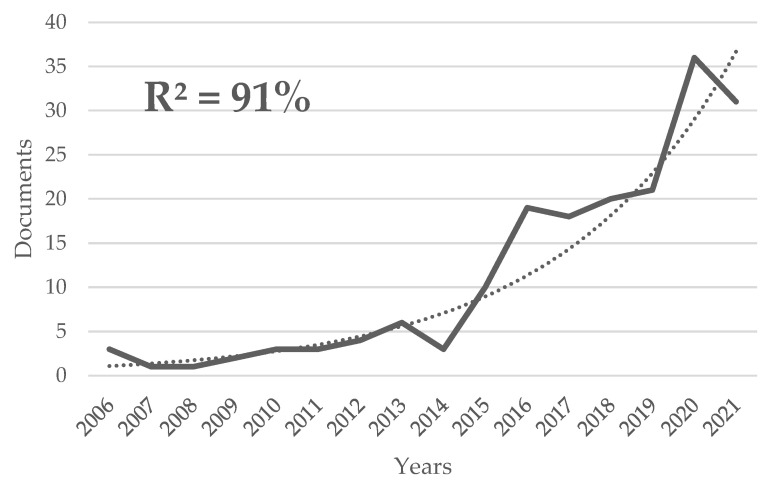
Annual publications trend on nature-based therapy research. The bold line represents the number of publications in each year, and the dotted one is the exponential growth curve that fits the number of publications.

Initially, 539 documents were obtained after the search. Then, a thorough review of titles, abstracts, and keywords was carried out by the researchers YRR and ADZ, excluding 301 papers, as they were related to fields different from nature-based therapy. Disagreements between reviewers were resolved via discussion, with 238 papers (211 articles and 27 reviews) published between 1937 to 2022 being finally selected (the full dataset is provided in Document S1 as Supplementary Material). An interrupted continuity was found between 2006 and the present (2022 was not considered, as it was not finished when the analyses were performed). The annual trend of publications adjusted 91% (R^2^) to an exponential growth ratio (Figure 1).

### 3.2. WoS Categories

The WoS category with the most related publication numbers was *Public, Environmental, and Occupational Health* (38 papers), followed by *Environmental Sciences* (33 papers); *Psychology, Clinical* (30 papers); and *Environmental Studies* and *Forestry* (20 papers each), these being the top five categories out of a total of 57 in which the WoS classified the documents (Table 1). In sixth place were *Psychiatry*, and *Rehabilitation* (both with 18 papers), followed by *Psychology Multidisciplinary* (17 papers), *Education and Educational Research* (15 papers), and *Plant Sciences* (13 papers).

### 3.3. Publications Titles

Documents were published in 137 journals with a publication number ranging from 1 to 33 publications. When applying Bradford’s law for the most important journals according to the number of publications, these were only distributed in two zones: Core and Zone I, which did not fit with Bradford’s model. The Core was composed of seven journals totalling 32.8% of the total number of publications and including journals that published at least six papers. Table 2 shows the core journals. The core journal was *The International Journal of Environmental Research and Public Health* (33 papers), *Urban Forestry & Urban Greening* (13 papers) and the *Journal of Adventure Education and Outdoor Learning* (7 papers). Bradford’s Zone I included the rest of the journals (130), totalling 67.2% of the total number of publications and a publication range between 1 and 4 papers. Since there were discrete values, it was impossible to establish an objective cut-off between zones I and II.

According to the number of citations, journals were distributed as follows: Core (2 journals), Zone I (12 journals), and Zone II (123 journals), with an error ratio of −9.7% over Bradford’s theoretical series. Core journals accounted for 32.9% of the total citations. The most cited journal was *The International Journal of Environmental Research and Public Health* with 33 papers and 824 citations. These were the only five journals with a total number of citations above 100. Table 3 shows Bradford’s Core and Zone I for the most cited journals. Table S1 provides information about Bradford’s Zones and their number of journals, based on the number of documents and cites.

### 3.4. Prolific and Influential Co-Authors

A total of 723 researchers were found in co-authorships in the analysed documents, with 85.8% submitting a single paper (620 co-authors); 8.4%, 2 publications (61 co-authors); and 5.9%, 3 or more (43 co-authors). Nine was the maximum number of publications found. According to Lotka, the prolific co-authors should be the 27 with the most publications (square root of 729). Since 17 authors were found with 4 or more publications and 43 with 3 or more, these 17 were the prolific. The most prolific co-authors were Nevin J. Harper and Anita R. Tucker (nine documents) from the University of Victoria (Saanich, BC, Canada) and the University of New Hampshire (Durham, NH, USA), respectively. Applying the h-index to the 17 prolific co-authors, authors with an average of 30 or more citations were estimated as prominent authors (the calculation of h-index will be detailed in Section 3.7). Table 4 shows the most prolific and prominent co-authors, their affiliations, locations, and the number of documents and citations.

**Figure 2:**
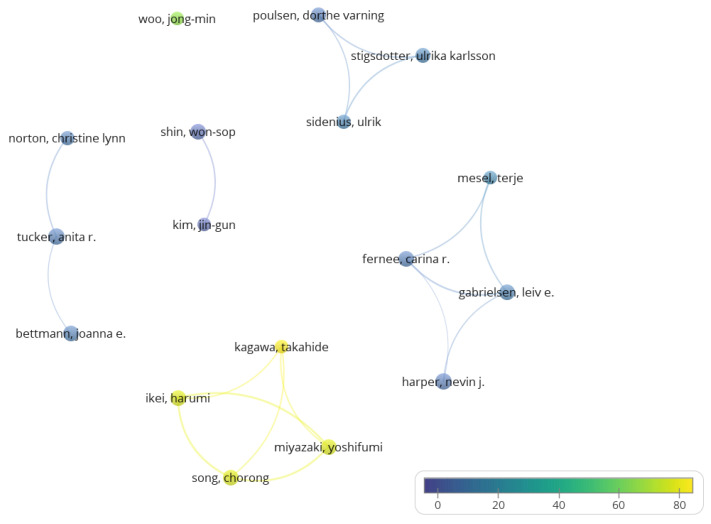
Prolific and prominent co-authors. VOSviewer: node size (documents); colour (average of citations). Notes: analysis: association strength; attraction: 10; repulsion: −4; scale: 1.35; bode size: documents; colour: citations average.

Figure 2 shows the 17 prolific co-authors and their interactions in the publications analysed. Six collaboration clusters emerged among the prominent co-authors, highlighting the cluster composed of Ikei, Kagawa, Miyazaki, and Song, with many manuscripts and citations.

### 3.5. Countries/Regions

Publication co-authors were found to come from 41 different countries/regions. The USA (61 papers) was the country with the highest number of publications, followed by South Korea (27 papers), Australia (19 papers), Canada (17 papers), and Norway (15 papers). Regarding citations, the USA again ranked first with 782 citations, followed by South Korea (701 citations), Japan (635 citations), Sweden (296 citations), and Norway and England (183 citations each).

**Figure 3:**
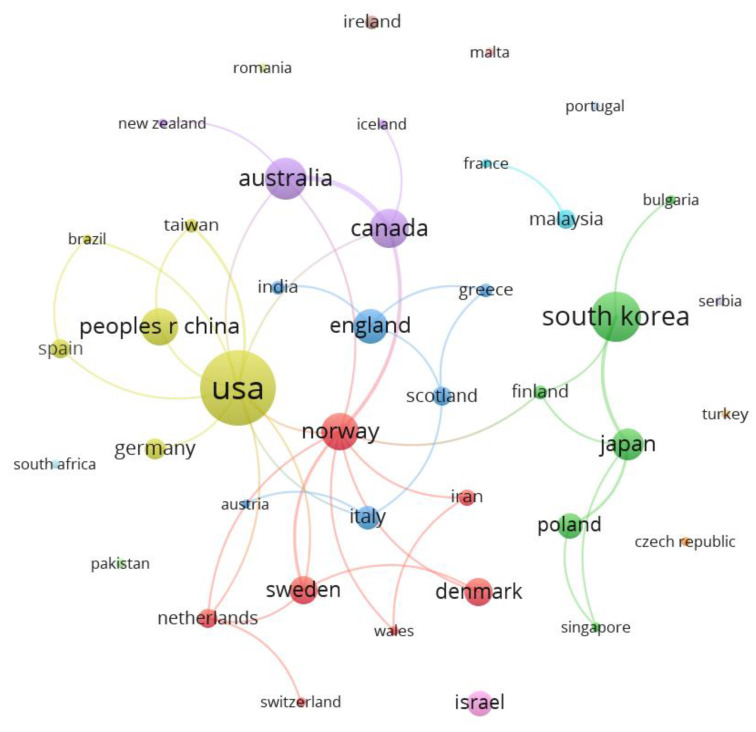
Countries/regions in co-authorship. VOSviewer: analysis: association strength; attraction: 6; repulsion: −2; clustering: resolution (1) and minimum cluster size (1); scale: 1.70; node size: documents; colour: cluster.

The regions/countries with the largest number of connections were the USA and Norway with 12 connections, followed by the Netherlands, Australia, Canada, Japan, and Sweden with 4 connections each. Figure 3 shows the co-authored countries/regions together with their connections, resulting in 17 clusters. The cluster with the highest number of publications was led by the USA and included Spain, Brazil, Germany, China, and Taiwan (yellow). The groups with the largest number of components were composed of Austria, England, Greece, India, Italy, and Scotland (blue) and the group composed of South Korea, Singapore, Poland, Japan, Finland, and Bulgaria (green), in addition to the one mentioned as the most productive, led by the USA. There is a five-component group consisting of Denmark, Iran, Norway, Sweden, and Wales (red), and a four-component group consisting of Australia, Canada, Iceland, and New Zealand (purple). The rest of the groups were composed of one or two components.

It is interesting to highlight that most papers related to forest therapies were published by researchers from countries such as China, Japan, and South Korea, but papers related to wilderness therapies were published mostly by researchers from the USA, along with Canada and Norway.

### 3.6. Author Keywords

**Figure 4:**
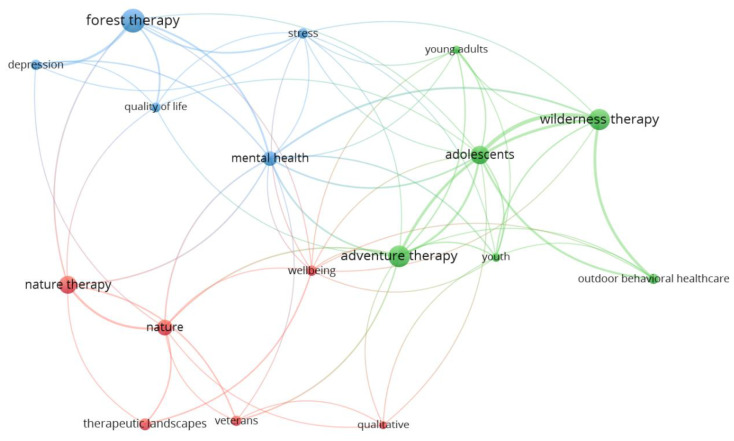
Most frequent author keywords. VOSviewer; analysis: association strength; attraction: 6; repulsion: −2; clustering: resolution (1) and minimum cluster size (1); scale: 1.70; node size: documents; colour: cluster.

The keywords used by the co-authors totalled 651 concepts. Following Zipf’s law, it was estimated that the most frequent keywords should be a number less or equal to 25. Moreover, 26 words with 4 or more occurrences and 17 words with 5 or more occurrences were found, the latter being considered the most relevant keywords. The keywords with the most occurrences were forest therapy (41 occurrences), adventure therapy (34 occurrences), wilderness therapy (33 occurrences), nature therapy (24 occurrences), and nature (18 occurrences). Regardless of the words used in the search mentioned above, the most frequent were adolescents (25 occurrences); mental health (16 occurrences); therapeutic landscapes (11 occurrences); and depression, outdoor behavioural healthcare, stress, veterans, and wellbeing with 8 occurrences each. Figure 4 shows the most frequently used keywords and how they were related to each other in the analysed publications, as well as the groupings between them. There were three well-defined clusters, a first cluster composed of six keywords with the words wilderness therapy and adventure therapy highlighted in green; a second cluster composed of another six keywords and headed by the term nature therapy highlighted in red; and a last cluster composed of four words highlighted in blue with the term forest therapy, which also includes the terms forest bathing and shinkin-yoku.

### 3.7. Documents

**Figure 5:**
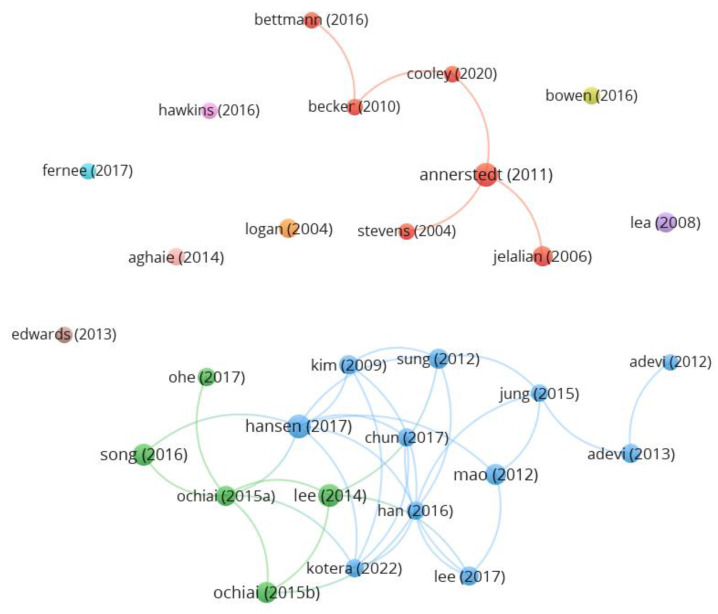
Most cited documents and their interrelationships. VOSviewer; analysis: association strength; attraction: 6; repulsion: −1; clustering: resolution (1) and minimum cluster size (1); scale: 1.35; node size: citations; colour: cluster.

A total of 29 documents were found with more than 30 citations, which were considered the most cited on the topic. The most cited document was “Shinrin-yoku (Forest Bathing) and Nature Therapy: A State-of-the-Art Review” by Hansen et al. (2017) [14] with 195 citations, published in *The International Journal of Environmental Research and Public Health* with the keywords shinrin-yoku, forest bathing, nature therapy, and integrative medicine. Figure 5 represents the most cited articles and their interrelationships, and Table S2 includes the full list of the most cited documents.

## 4. Discussion

This bibliometric analysis was the first in mapping publications on nature-based therapy, covering 238 documents. The annual publication trend on the subject followed an exponential growth rate between 2006 and 2021, with 198 publications in that period, and high growth in the last 2 years. This finding highlights the interest of the international scientific community in this topic [60]. Despite exponential growth in recent years, articles have been published since 1937, but before 2006, there was no continuity in annual publications, with a total of 24 articles published between 1937 and 2004 (publications in discontinuous years), which is approximately 8 times less than what was published in the last 16 years. The first article published on the subject, “The Significance of the Forest for the Therapeutic Value of the German Low Mountain Range”, was published in 1937 [61]. A similar bibliometric study was found for publications related to forest therapy [44], but it only considered the period between 2007 and 2021 and did not apply the traditional laws of bibliometrics [48,49,51,52,55,59]. The existence of various bibliometrics with similar themes indicates an interest in the field, in line with the increase in the number of researchers interested in nature-based therapies as a means of escaping from urban routines. This research also identified the core journals and most cited journals, highlighting the most productive (prolific) and prominent authors, the most relevant articles, and the keywords most used by researchers, offering useful information for those interested in the subject, helping to find researchers or journals involved in this field of study as well as potential collaborations. Figure 1 shows the increase in scientific production in this area starting in 2020. It is unknown whether this could be related to the COVID-19 pandemic, based on the need for outdoor activities or as a response to population confinement and/or the psychological problems that this entailed. Other bibliometrics on similar topics also reported a massive increase in publications in 2020 [44]. Among the findings of this study was that forest and adventure therapies were of greater interest to researchers than other nature-based therapies, according to the number of publications related to and keywords in the set of documents. Thus, landscape or coastal therapies, among others, appear to be less researched approaches or of more recent interest, turning into new lines of research on the treatment of mental health and wellbeing.

When analysing journal distribution according to the number of documents, Bradford’s theoretical distribution was not followed: only 7 formed the Core, accumulating 33% of the publications (between 4 and 33 publications per journal), and 130 journals formed Zone I, totalling 67% of the publications (between 1 and 3 publications), making it impossible to distribute the journals in 3 zones, each accounting for 33% of the publications. This may be due to the lack of a high volume of journals specific to the subject or those with a high interest in it. In contrast, a high volume of journals with low interest in the subject area was found, reflected by the fact that more than half of the journals (120 out of 238, 50.4%) published only 1 paper on the subject (120 journals with only 1 paper). As shown in Figure 1, this can be considered an object of study of growing interest, with the number of publications currently increasing, which indicates that there is not yet a high number of journals with an interest in this topic. According to the results, two journals appeared to stand out regarding the subject, attracting not only the highest number of papers but also the highest number of citations, with *The International Journal of Environmental Research and Public Health* (MDPI) and *Urban Forestry & Urban Greening* (Elsevier GMBH) also being two journals with a high impact factor, both in Quartile 1 (2022) of their respective categories. The core journal outnumbered the second-ranked journal by 20 publications and was the reference journal for the submission of studies on nature-based therapies for researchers.

The WoS category with the most publications was “Public, Environmental, and Occupational Health” with 38 documents, with the journal with the most publications on the subject being *The International Journal of Environmental Research and Public Health*, with 29 publications in this category. Within this WoS category, the most common topics were forest therapy, forest baths, and shinrin-yoku. This category also contains the first 2 most cited articles, both with more than 100 citations: “Shinrin-Yoku (Forest Bathing) and Nature Therapy: A State-of-the-Art Review” by Hansen et al. (2017) [14] and “Nature-Assisted Therapy: Systematic Review of Controlled and Observational Studies” by Annerstedt and Wahrborg (2011) [62]. “Environmental Sciences” was the next most cited subject category with 33 papers. Among the most cited papers, there were 6 with more than 100 citations, most of them having already been discussed: the first was “Shinrin-Yoku (Forest Bathing) and Nature Therapy: A State-of-the-Art Review” by Hansen et al. (2017) with 195 citations [14]; the second was “Nature-Assisted Therapy: Systematic Review of Controlled and Observational Studies” by Annerstedt and Wahrborg (2011) [63]. Third, “Influence of Forest Therapy on Cardiovascular Relaxation in Young Adults” by Lee et al. (2014), published in *Evidence-Based Complementary and Alternative Medicine*, focused on elucidating the health benefits of forest walking on cardiovascular reactivity, with 147 citations [63]. All the highlighted papers were relatively modern, from the 20th century, so there is no classic main reference document for researchers in the field. Although adventure and wilderness seem to draw more interest than forest therapies according to the number of publications or keywords used by authors, forest therapies seem to have a higher citation power.

Figure 4 shows the most frequently used keywords and their joint use by the co-authors. Different interpretations can be made about the themes of the different therapeutic approaches that generated the most research interest. Three main groupings were identified: the first (blue) was headed by the keywords “forest therapy” and used by the authors together with “depression”, “stress”, “quality of life”, and “mental health”, evidencing that some of the research trends related to forest therapy, mental health, and quality of life are popular research topics for researchers in this topic of study. The second grouping (green) was led by “wilderness therapy” and “adventure therapy” and was related to young adults, adolescents, youth, and outdoor behavioural healthcare. This could establish a trend in the research on these therapies, especially studies focused on the young population. Such a population may have a greater predisposition for more exciting activities, and adventurous therapies may be more attractive to this type of population by provoking more powerful stimuli that bring benefits to its physical and mental health. In these two main clusters, the keywords most used by the co-authors were grouped, coinciding with the therapies of greatest interest to researchers according to the number of articles published related to them, reinforcing one of the findings of this study: forest therapy, adventure therapy, and wilderness therapy have higher scientific production than other therapies carried out in nature. The third relevant cluster (red) was headed by “nature therapy”, accompanied by keywords such as “nature”, “wellbeing”, “veterans”, “therapeutic landscapes”, and “qualitative”; this cluster presented a lower number of connections and grouped terms with a lower occurrence of use, including therapies with a lower number of publications that seem to be currently developing.

Considering the results obtained from the distribution of publications by country, the USA emerged as the country with the highest number of publications, in line with the findings of other bibliometric studies on the subject [44], as it is a country with a large number of researchers who increase scientific production in most fields [64,65,66,67]. The USA doubled the number of publications of the second most prolific country, South Korea, followed by Australia and Canada, with China in the sixth position, in contrast to a study related to forest therapies, where it was the second most prolific country. In this regard, an uneven geographical distribution was found according to the study of the different approaches: forest therapies were linked more to oriental countries (North Korea, China, and Japan), while adventure or wilderness therapies had more interest in countries such as the USA, Canada, and Norway. When looking at countries that stood out as the most influential, the USA, South Korea, and Japan ranked in the top three, with a difference of more than 300 citations compared with Sweden’s fourth position.

Nevin J. Harper (Canada) and Anita R. Tucker (USA) were the prolific authors with 9 papers each, although they were not the most citated (78 and 92 citations, respectively). Harper had connections with three authors with affiliations in Norway: Fernee, Gabrielsen, and Mesel. The first two stand out in the number of publications (8 papers), but not in the number of citations, Gabrielsen being the most influential in this cluster with 100 citations. The second, much more influential cluster, featuring Ikei, Miyazaki, and Song, with 8 papers and 597 citations, was connected to the fourth most influential author, Kagawa, with 4 papers and 382 citations. This cluster explored the applications and characteristics of forest therapy, which is also highlighted by bibliometrics in this area [44]. This cluster of researchers is the most influential in forest therapies [7]. Nevin J. Harper collaborated with three authors with affiliations in Norway, C. R. Fernee, L. E. Gabrielsen, and T. Mesel, the first two of whom stood out in terms of the number of publications (8 papers), but not in citations, with L. E. Gabrielsen being the most influential in this cluster with a total of 100 citations. These co-authors produced papers on adventure therapies, suggesting that they may be less cited publications than those related to forest therapies. The other prolific co-author, Anita R. Tucker, had collaborations with Joanna E. Bettmann (8 publications) and Christine Lynn Norton (5 papers), both from the USA, both with a lower number of citations than the next publication cluster. The third publication cluster was formed by the 3 most prominent co-authors, H. Ikei, Y. Miyazaki, and C. Song (8 papers, 597 citations, and 5 highly cited papers), who all have a connection with the fourth most influential author, T. Kagawa (4 papers, 382 citations, and 3 highly cited papers), all published on forest therapies. These co-authors explored the applications and characteristics of forest therapies in the population, as highlighted by specific bibliometrics in that area [44]. This group of researchers is the most influential in the area of forest therapies, despite being a current group that is still active and that published its last article on the subject in 2020 [7]. In addition, the prominent co-author Yoshifumi Miyazaki is a reference in the field, authoring several books on forest baths, which were not included in this analysis, as this was not the purpose of this study. Similarly, the prolific authors, Nevin J. Harper and Anita R. Tucker, were also co-authors of other types of publications, not included in this analysis because they are not included in journals indexed in WoS or they are published in different formats.

This bibliometric review reveals the increasing attention paid by journals, publishers, and editors to nature-based therapies, as well as the number of researchers publishing on the topic. Moreover, it provides a foundation for advancing knowledge and research in this field, suggesting that articles on the topic could receive a significant amount of attention. Thus, this study displays relevant information on researchers interested in the subject and serves as a reference for further research in the field. Furthermore, it can be used to assess the quality and impact of research on society and policymaking.

The limitations of this study are related to the possibility of having experienced publication bias since, despite having used one of the most complete, prestigious, and widely used databases for bibliometric studies (the WoS), a search was not carried out in other databases. Therefore, it can be assumed that documents published in journals that are not indexed in the WoS or with a lower impact may have been omitted. Future research may consider complementary research using other databases such as Scopus or PubMed. Moreover, it would be interesting to consider bibliometric analyses focusing on the type of therapy being analysed, such as forest therapy or blue therapy, in future research.

## 5. Conclusions

Nature-based therapies are applied in different variants to diverse diseases and conditions, highlighting their applicability regardless of the psychological or mood problems. Annual publications on nature-based therapies have shown an exponential growth trend in recent years, including a large base of researchers, journals, and publishers involved in the topic. The most researched keywords included forest baths and wilderness therapy. Moreover, forest therapy was mainly focused on treating mental health aspects, while wilderness therapy is mainly used with adolescents and the young population, showing a connection between natural therapies and the term “mental health”. The core journals in the field were *The International Journal of Environmental Research and Public Health* (MDPI) and *Urban Forestry & Urban Greening* (Elsevier GMBH). The most prominent research group was the one formed by Ikei, Miyazaki, and Song, and the USA was the country with the largest number of manuscripts and citations.

## Figures and Tables

**Table 1:** Top five Web of Science (WoS) categories according to the number of indexed documents.

Web of Science Categories	Number of Documents
Public, Environmental, and Occupational Health	38
Environmental Sciences	33
Psychology, Clinical	30
Environmental Studies	20
Forestry	20

**Table 2:** Bradford’s Core: most important journals.

Journals (Publisher)	Docs	%Docs	Cites	JIF/JCI	Q	%OA
*International Journal of Environmental Research and Public Health* (MDPI)	33	13.9	824	4.614 (JIF)	Q1	95
*Urban Forestry & Urban Greening* (Elsevier GMBH)	13	5.5	293	5.766 (JIF)	Q1	10.2
*Journal of Adventure Education and Outdoor Learning* (Routledge Journals, Taylor & Francis)	7	2.9	22	0.97 (JCI)	Q2 (JCI)	15.8
*Journal of Experiential Education* (Sage Publications)	7	2.9	51	0.99 (JCI)	Q2 (JCI)	7
*Contemporary Family Therapy* (Springer)	6	2.5	43	0.36 (JCI)	Q3 (JCI)	4.8
*Frontiers in Psychology* (Frontiers Media)	6	2.5	6	4.232 (JIF)	Q1	99.5
*Women & Therapy* (Routledge Journals, Taylor & Francis)	6	2.5	63	1.484 (JIF)	Q3	1.2

Docs: documents; %Docs: documents percentage; JIF: Journal Impact Factor; JCI: Journal Citation Indicator for ESCI Journals; Q: Journal Citation Report Quartile; %OA: Open-Access percentage.

**Table 3:** Bradford’s Core and Zone 1 most cited journals.

Bradford’s Zones	Journals (Publishers)	Docs	Cit	%Cit	JIF	Q.	%OA
Core	*International Journal of Environmental Research and Public Health* (MDPI)	33	824	23.4	4.614	Q1	95
	*Urban Forestry & Urban Greening* (Elsevier GMBH)	13	293	8.3	5.766	Q1	10.2
Zone I	*Scandinavian Journal of Public Health* (Sage Publications)	1	171	4.9	3.199	Q2	33.2
	*Evidence-Based Complementary and Alternative Medicine* (Hindawi)	1	147	4.2	2.650	Q3	98.7
	*Journal of Cardiology* (Elsevier)	1	103	2.9	2.974	Q3	8
	*Child & Youth Care Forum* (Springer)	4	91	2.6	2.203	Q3	18.8
	*International Journal of Obesity* (Springer Nature)	1	91	2.6	5.551	Q2	23
	*Clinical and Experimental Hypertension* (Taylor & Francis)	1	90	2.6	2.088	Q4	3.8
	*Area* (Wiley)	1	83	2.4	2.057	Q3	24.9
	*BMJ—British Medical Journal* (BMJ Publishing Group)	1	77	2.2	93.467	Q1	81.9
	*Journal of Child and Family Studies* (Springer)	3	71	2.0	2.784	Q2	10.8
	*Psychiatry Investigation* (Korean Neuropsychiatric Assoc)	1	67	1.9	3.202	Q3	99.5
	*Women & Therapy* (Routledge Journals, Taylor & Francis)	6	63	1.8	1.484	Q3	1.2
	*International Journal of Neuroscience* (Taylor & Francis)	1	62	1.8	2.590	Q4	2.1

Docs: documents; Cit: number of citations; %Cit: citations percentage; JIF: Journal Impact Factor; JCI: Journal Citation Indicator; Q: Journal Citation Report Quartile; *; %OA: Open-Access percentage.

**Table 4:** Prolific and prominent co-authors.

Co-authors (Most Cited Documents)	Affiliation/Country-Region	Documents	Citations	Cit/Doc
Ikei, Harumi *	Chiba University/Japan	8	597	75
Miyazaki, Yoshifumi *	Chiba University/Japan	8	597	75
Song, Chorong *	Kongju National University/South Korea	8	597	75
Kagawa, Takahide *	Chiba University/Japan	4	382	96
Woo, Jong-Min *	Inje University/South Korea	4	251	63
Gabrielsen, Leiv E.	Sorlandet Sykehus/Norway	8	100	13
Tucker, Anita R.	University of New Hampshire/USA	9	92	10
Bettmann, Joanna E.	University of Utah/USA	8	87	11
Fernee, Carina R.	University of Agder/Norway	8	78	10
Harper, Nevin J.	University of Victoria/Canada	9	78	9
Stigsdotter, Ulrika Karlsson	University of Copenhagen/Denmark	6	76	13
Sidenius, Ulrik	University of Copenhagen/Denmark	5	68	14
Poulsen, Dorthe Varning	University of Copenhagen/Denmark	7	64	9
Mesel, Terje	University of Agder/Norway	4	63	16
Norton, Christine Lynn	Texas State University System/USA	5	55	11
Shin, Won-Sop	Chungbuk National University/South Korea	8	42	5
Kim, Jin-Gun	Chungbuk National University/South Korea	4	19	5

* Prominent co-authors. Prolific co-authors with documents among most cited papers.

## Data Availability

Datasets are available upon reasonable request.

## Supplementary Materials

The following supporting information can be downloaded at https://www.mdpi.com/article/10.3390/healthcare11091249/s1: Document S1: Full dataset in plain text. Table S1. Bradford’s zones and their number of journals according to the number of documents and cites. Table S2. Most cited documents.
