# Supplementary material for: Bibliometric Analysis of Nature-Based Therapy Research
Source: Healthcare (Basel). 2023 Apr 27;11(9):1249. doi: 10.3390/healthcare11091249 (PMC10178870; doi:10.3390/healthcare11091249)
Supplement: Supplementary file 1 [file healthcare-11-01249-s001.zip › SUPP Table S1.pdf]

Table S1. Bradford's zones and their number of journals, according to the number of documents and cites.

According to number of documents

Zone	Nº Journals (%)	Number Articles (%)	Acc. Nº Journals (%)	Acc. Nº Articles (%)	Bradford multipliers	Journals (theoretical serie)	
Core	7 (5%)	78 (33%)	7 (5%)	78 (33%)		n0	7
Zone 1	130 (95%)	160 (67%)	137 (100%)	238 (100%)	18.6	n1	65
Zone 2	0 (0%)	0 (0%)	137 (100%)	238 (100%)	0.0	n2	604
Total	137 100%	238 100%			Mean 9.3		676
						Error	-393.1%

According to number of cites.

Zone	Nº Journals (%)	Number Articles (%)	Nº Citations (%)	Acc. Nº Citations (%)	Bradford multipliers	Journals (theoretical serie)	
Core	2 (1%)	46 (19%)	1117 (33%)	1117 (33%)		n0	2
Zone 1	12 (9%)	22 (9%)	1116 (33%)	2223 (66%)	6.0	n1	16
Zone 2	123 (90%)	170 (71%)	1165 (34%)	3398 (100%)	10.3	n2	132
Total	137 100%	238 100%	3398 (100%)		Mean 8.1		259
						Error	-9.7%

Nº (Number); % (Percentage); Acc. (Accumulated).
