# Supplementary material for: Bibliometric Analysis of Nature-Based Therapy Research
Source: Healthcare (Basel). 2023 Apr 27;11(9):1249. doi: 10.3390/healthcare11091249 (PMC10178870; doi:10.3390/healthcare11091249)
Supplement: Supplementary file 1 [file healthcare-11-01249-s001.zip › SUPP Table S2.pdf]

Table S2. Most cited documents.

| Title. Main author (Year of publication) | Journal ISO Abbreviation | Cites |
|---|---|---|
| Shinrin-Yoku (Forest Bathing) And Nature Therapy: A State-Of-The-Art Review. Hansen et al. (2017) | Int. J. Environ. Res. Public Health | 195 |
| Nature-Assisted Therapy: Systematic Review Of Controlled And Observational Studies. Annerstedt y Wahrborg (2011) | Scand. J. Public Health | 171 |
| Influence Of Forest Therapy On Cardiovascular Relaxation In Young Adults. Lee et al. (2014) | Evid.-based Complement Altern. Med. | 147 |
| Physiological Effects Of Nature Therapy: A Review Of The Research In Japan. Song et al. (2016) | Int. J. Environ. Res. Public Health | 131 |
| Physiological And Psychological Effects Of Forest Therapy On Middle-Aged Males With High-Normal Blood Pressure. Ochiai et al. (2015) | Int. J. Environ. Res. Public Health | 112 |
| Therapeutic Effect Of Forest Bathing On Human Hypertension In The Elderly. Mao et al. (2012) | J. Cardiol. | 103 |
| Physiological And Psychological Effects Of A Forest Therapy Program On Middle-Aged Females. Ochiai et al. (2015) | Int. J. Environ. Res. Public Health | 94 |
| Adventure Therapy' Combined With Cognitive-Behavioral Treatment For Overweight Adolescents. Jelalian et al. (2006) | Int. J. Obes. | 91 |
| The Effect Of Cognitive Behavior Therapy-Based Forest Therapy Program On Blood Pressure, Salivary Cortisol Level, And Quality Of Life In Elderly Hypertensive Patients. Sung et al. (2012) | Clin. Exp. Hypertens. | 90 |
| Retreating To Nature: Rethinking 'Therapeutic Landscapes'. Lea, J. (2008) | Area | 83 |
| Randomised Controlled Trial Of An Occupational Therapy Intervention To Increase Outdoor Mobility After Stroke. Logan et al. (2004) | BMJ-British Medical Journal | 77 |
| Effects Of Forest Therapy On Depressive Symptoms Among Adults: A Systematic Review. Lee et al. (2017) | Int. J. Environ. Res. Public Health | 73 |
| Stress Rehabilitation Through Garden Therapy: The Garden As A Place In The Recovery From Stress. Adevi y Martensson (2013) | Urban For. Urban Green. | 70 |
| The Effect Of Cognitive Behavior Therapy-Based Psychotherapy Applied In A Forest Environment An Physiological Changes And Remission Of Major Depressive Disorder. Kim et al. (2009) | Psychiatry Investig. | 67 |
| The Effects Of Forest Therapy On Depression And Anxiety In Patients With Chronic Stroke. Chun et al. (2017) | Int. J. Neurosci. | 62 |
| Evaluating The Relaxation Effects Of Emerging Forest-Therapy Tourism: A Multidisciplinary Approach. Ohe et al. (2017) | Tourism Manage. | 53 |
| Effects Of Shinrin-Yoku (Forest Bathing) And Nature Therapy On Mental Health: A Systematic Review And Meta-Analysis. Kotera et al. (2022) | Int. J. Mental Health Addict. | 52 |
| The Effects of Forest Therapy on Coping with Chronic Widespread Pain: Physiological and Psychological Differences between Participants in a Forest Therapy Program and a Control Group | Int. J. Environ. Res. Public Health | 50 |
| Effect of a forest therapy program and the forest environment on female workers' stress. Jung et al. (2015) | Urban For. Urban Green. | 44 |
| Effect of nature-based sound therapy on agitation and anxiety in coronary artery bypass graft patients during the weaning of mechanical ventilation: A randomised clinical trial. Aghaie et al. (2014) | Int. J. Nurs. Stud. | 43 |
| Wilderness adventure therapy effects on the mental health of youth participants. Bowen et al. (2016) | Eval. Program Plan. | 42 |
| Wilderness Therapy: Ethical Considerations for Mental Health Professionals. Becker et al. (2010) | Child Youth Care Forum | 38 |
| A Meta-analysis of Wilderness Therapy Outcomes for Private Pay Clients. Bettmann et al. (2016) | J. Child Fam. Stud. | 37 |
| An evaluation of a therapeutic garden's influence on the quality of life of aged care residents with dementia. Edwards et al. (2013) | Dement.-Int. J. soc. Res. Pract. | 35 |

| | | |
|---|---|---|
| Stress rehabilitation through garden therapy A caregiver perspective on factors considered most essential to the recovery process. Adevi et al. (2012) | Urban For. Urban Green. | 34 |
| Adventure therapy for adolescents with cancer. Stevens et al. (2004) | Pediatr. Blood Cancer | 33 |
| Nature-Based Recreational Therapy for Military Service Members A Strengths Approach. Cooley et al. (2020) | Ther. Recreat. J. | 33 |
| Into the Wild': A meta-synthesis of talking therapy in natural outdoor spaces. Hawkins et al. (2016) | Clin. Psychol. Rev. | 33 |
| Unpacking the Black Box of Wilderness Therapy: A Realist Synthesis. Fernee et al. (2017) | Qual. Health Res. | 32 |

Cites (Times cited in Web of Science Core).
